# The impact of experiencing severe physical abuse in childhood on adolescent refugees’ emotional distress and integration during the COVID-19 pandemic

**DOI:** 10.3389/fpsyg.2022.1023252

**Published:** 2022-11-24

**Authors:** Flurina Potter, Katalin Dohrmann, Brigitte Rockstroh, Maggie Schauer, Anselm Crombach

**Affiliations:** ^1^Department of Psychology, University of Konstanz, Konstanz, Germany; ^2^Department of Psychology, University of Saarbrücken, Saarbrücken, Germany

**Keywords:** refugees, physical abuse, childhood abuse, post-migration stressors, COVID-19 pandemic integration, emotional distress, mental health

## Abstract

**Background:**

Accumulating evidence highlights the importance of pre- and post- migration stressors on refugees’ mental health and integration. In addition to migration-associated stressors, experiences earlier in life such as physical abuse in childhood as well as current life stress as produced by the COVID-19-pandemic may impair mental health and successful integration – yet evidence on these further risks is still limited. The present study explicitly focused on the impact of severe physical abuse in childhood during the COVID-19 pandemic and evaluated the impact of these additional stressors on emotional distress and integration of refugees in Germany.

**Methods:**

The sample included 80 refugees, 88.8% male, mean age 19.7 years. In a semi-structured interview, trained psychologists screened for emotional distress, using the Refugee Health Screener, and integration status, using the Integration Index. The experience of severe physical abuse in childhood was quantified as a yes/no response to the question: “Have you been hit so badly before the age of 15 that you had to go to hospital or needed medical attention?” Multiple hierarchical regression analyses further included gender, age, residence status, months since the start of the COVID-19 pandemic and length of stay in Germany to predict emotional distress and integration.

**Results:**

Two regression analyses determined significant predictors of (1) emotional distress (adjusted *R*^2^ = 0.23): duration of being in the pandemic (*ß* = 0.38, *p* < 0.001) and severe physical abuse in childhood (*ß* = 0.31, *p* = 0.005), and significant predictors of (2) integration (adjusted *R*^2^ = 0.53): length of stay in Germany (*ß* = 0.62, *p* < 0.001), severe physical abuse in childhood (*ß* = 0.21, *p* = 0.019) and emotional distress (*ß* = −0.28, *p* = 0.002).

**Conclusion:**

In addition to migration-associated stressors, severe physical abuse in childhood constitutes a pre-migration risk, which crucially affects the well-being, emotional distress and integration of refugees in Germany.

## Introduction

From 2010 to 2019 the number of refugees worldwide has doubled from about 10 million in 2010 to 20.4 million in 2019 (United Nations High Commissioner for Refugees, 2020). In 2021, the United Nations High Commission for Refugees estimated that the number of refugees worldwide was as high as 26.6 million and the number of asylum seekers^1^ as high as 4.4 million (United Nations High Commissioner for Refugees, 2021). In 2019, approximately half of the refugees were minors below 18 years of age and 13% were young adults between 18 and 24 years (United Nations High Commissioner for Refugees, 2020). Germany alone hosted 1.2 million refugees in 2021 (United Nations High Commissioner for Refugees, 2021) and accepted the largest number of asylum applications worldwide in the past decade (United Nations High Commissioner for Refugees, 2020; Hoell et al., 2021). The activities and efforts to enable social integration and support the refugees’ well-being also brought forth many challenges (Silove et al., 2017; Kaltenbach, 2019). For instance, a Swiss study showed that even after 10 years of residence in Switzerland, refugees showed serious integration difficulties, including struggling with language barriers, isolation and unemployment (Schick et al., 2016). An impairing factor may be that refugees from war- and/or violence-inflicted regions suffered from mental disorders resulting from these experiences more often than the adult population in Western nations (Hoell et al., 2021): Compared to the general adult population refugees are up to 15 times more likely to suffer from post-traumatic stress disorder (PTSD) and up to 14 times more likely to suffer from depression, prevalence rates varying between 4.4–86% for PTSD and 2.3–80% for depression (Bogic et al., 2015). A recent meta-analysis focusing on German refugees and asylum seekers indicated a prevalence of 29.9% for PTSD, and 39.8% for depression (Hoell et al., 2021). Similar if not higher prevalence rates were found in refugee youth. According to a recent review, half of the refugee youth might be affected by PTSD and up to a third by emotional or behavioral problems, such as depression or anxiety disorder (Kien et al., 2019).

### Pre-migration stressors

Different stressors experienced pre-migration, i.e., prior to leaving the home country, peri-migration, during the flight, and post-migration, upon arrival in the host country, have been shown to affect refugees’ mental health, making it even more likely for them to suffer and impair their integration (Schick et al., 2016; Kartal et al., 2018; Aragona et al., 2020). These stressors might affect refugee youth even more severely, as they simultaneously undergo immense physical and mental changes (Schneider et al., 2017). Many refugees have experienced **pre-migration stressors** such as atrocities during war and other traumatic events in their home country and/or on the journey to Europe (Davidson et al., 2010; Bajbouj et al., 2021). In the general population as well as in refugee populations, an elevated trauma load has been associated with an elevated risk for mental ill-health and stress related mental disorders (Steel et al., 2009; Wilker et al., 2015; Kartal et al., 2018). Among potentially traumatic events, interpersonal violence had the strongest associations with mental problems (Tinghög et al., 2017). In addition, reports and observations suggested that adverse experiences earlier in life and before war/crisis-associated traumata impair healthy development, increase the risk for mental health problems and the ability to flexibly adapt to new social situations (Felitti et al., 1998; Kendall-Tackett, 2002; Olema et al., 2014; Kalia and Knauft, 2020). Research has found that older minors were exposed to more traumatic experiences than younger minors (Bean et al., 2007), and that even when controlling for age unaccompanied refugee minors were exposed to more traumatic experiences than accompanied refugee minors (Müller et al., 2019; Müller-Bamouh et al., 2020). In particular, physical abuse and other types of abuse during childhood have been identified as increasing the risk for PTSD (Margolin and Vickerman, 2011), adult depression and anxiety (Lindert et al., 2014), and a more severe course of mental disorders (Teicher and Samson, 2013). High rates of child maltreatment have been reported in war-affected countries (Catani et al., 2008, 2009) and it was shown that the impact of maltreatment in childhood surpassed the damage of recent war trauma (Olema et al., 2014). In a national cohort study, Webb et al. (2017) examined the association of hospital admissions because of self-harm, accidents and interpersonal violence before the age of 15 and self-harm and violent offending at ages 15–35. About one in four men admitted to hospital because of interpersonal violence before the age of 15 was later convicted for committing a violent crime (Webb et al., 2017). **Severe physical abuse in childhood** leading to hospital admissions showed a significant prognostic value and Webb et al. (2017) stated that these results were probably internationally generalizable. Furthermore, interpersonal violence seemed to increase the risk for the perpetration of violence (Weaver et al., 2008), which might be a risk factor against successful reintegration.

### Post-migration stressors

Aiming to assess the unique impact of severe physical abuse in childhood on refugee’s emotional distress and integration, this study takes the influence of known post-migration stressors into account. Several **post-migration stressors** -including a long asylum procedure, lack of employment or limited access to health care- interact in their impact on the refugees’ mental health (Laban et al., 2005; Böttche et al., 2016; Schneider et al., 2017; Bauhoff and Göpffarth, 2018; Kaltenbach, 2019). In fact, numerous longitudinal studies have demonstrated that emotional distress and post-migration stressors seem to mutually reinforce each other (Bakker et al., 2014; Tingvold et al., 2015; Li et al., 2016). Examining adolescent refugees, it was found that even among severely traumatized youth, post-migration stressors were powerfully related to the presence of PTSD symptoms (Ellis et al., 2008). One of the most prominent post-migration stressors influencing the mental health of refugees is their asylum procedure and **residence status** (Baron and Flory, 2020). In a longitudinal study, the symptom severity of PTSD, anxiety and depression of refugees with accepted claims and refugees with rejected claims was examined (Silove et al., 2007). Despite similar pre-migration trauma and baseline psychiatric symptoms the symptom severity of refugees with accepted claims improved substantially, whilst refugees with rejected claims maintained high symptom severity levels (Silove et al., 2007). Further studies reported that accepted refugees showed less PTSD, anxiety and depression symptoms than asylum seekers (Gerritsen et al., 2006; Stenmark et al., 2013). Several studies suggested that the length of the asylum procedure severely affects mental health (Laban et al., 2005; Phillimore, 2011). Heeren et al. (2016) found that the association between residence status and depression and anxiety remained significant even after controlling for other influencing factors, such as traumatic events, integration and social desirability. Interestingly, in this research the diagnosis of PTSD was independent of the residence status (Heeren et al., 2016).

In addition to the stress related to the residence status, including the risk of being sent back to perilous living conditions in the country of origin, refugees must deal with acculturative stress whilst adapting to the new host culture (Berry, 2005; Phillimore, 2011; Kartal et al., 2018). Amongst four acculturation strategies: assimilation, marginalization, separation and integration, the latter is considered best for emotional well-being (Berry, 2005; Behrens et al., 2015; Han et al., 2016). In contrast to the other strategies, which resist engaging with either the culture of origin and/or the host culture, **integration** aims at maintaining the heritage culture whilst aspiring to become fully engaged in the host society (Han et al., 2016). Harder et al. (2018) defined integration “as the degree to which immigrants have the knowledge and capacity to build a successful, fulfilling life in the host society” (p.2). In this context knowledge refers to the comprehension of the host country’s political system, social institutions and national language coupled with the skill to navigate the labour market of the host country; whereas capacity in this context refers to mental, economic and social assets immigrants have to invest in their futures (Harder et al., 2018). Integration is, in this definition, subdivided into six different dimensions: psychological, economic, political, social, linguistic as well as navigational integration (Harder et al., 2018). Research has demonstrated that emotional distress of refugees impaired their integration process. Phillimore (Phillimore, 2011) noted that refugees diagnosed with mental disorders struggled to engage in integrating activities, such as seeking employment or forming relationships with the host population. In longitudinal studies, increased psychological distress 3 years post-migration predicted acculturative difficulties 23 years post-migration (Tingvold et al., 2015). However, the success of integration also influences emotional distress. A longitudinal study on refugees showed that post-migration stressors such as cultural integration, financial stress and loneliness -over and above pre-migration stressors- affected refugees’ mental health and disrupted their recovery of mental health over the course of resettlement of 5 years (Stuart and Nowosad, 2020). It has been proposed that pre-migration stressors, post-migration stressors and fear for the future create an ongoing “continuum of stress” for refugees (Silove et al., 1991; Nickerson et al., 2011).

### Covid-19 pandemic

On top of all these already examined post-migration stressors, a new additional potential challenge of well-being and mental health has evolved with the **Covid-19 pandemic.** Indeed, the Covid-19 pandemic has already been reported to affect psychiatric disorders (Alpay et al., 2021; Taquet et al., 2021) and even exacerbate PTSD symptoms due to isolation, loss of control and the experience of repeated helplessness (Kizilhan and Noll-Hussong, 2020; Mattar and Piwowarczyk, 2020; Rees and Fisher, 2020). It has already been suggested that the negative consequences of the pandemic may affect vulnerable groups such as refugees even more than the general population (Aragona et al., 2020; Alpay et al., 2021; Gibson et al., 2021). Yet, evidence delineating the potential stressful impact of the Covid-19 pandemic on the mental health of refugees is still limited (Bernardi et al., 2021).

### Aim of this study

Considering the above cited evidence, the present study emphasized the following stress factors as predictors of emotional distress and successful integration in a sample of adolescent refugees in Germany: severe physical abuse in childhood and current Covid-19 pandemic.

## Materials and methods

### Sample

Altogether 97 refugees were recruited if they met the inclusion criteria of refugee status and presenting sufficient understanding of procedures and questions. Data of *n* = 12 refugees had to be excluded due to too much missing data on one of the key variables: severe physical abuse in childhood, emotional distress or integration. Data of one refugee aged 41 years was excluded due to the research’s focus on refugee adolescents. Moreover, as physical abuse experienced before the age of 15 years was determined as index of severe physical abuse in childhood, *n* = 4 refugees younger than 15 years had to be excluded. The final sample of *N* = 80 included 71 male (88.8%) and 9 female (11.3%) participants, aged 15 to 27 years, mean age 19.7 years (*SD* = 2.2 years). In case of missing socio-demographic data, missing values are indicated by the deviating *n* in the results section.

### Design and procedure

The assessment was part of a larger project implemented by the Center of Excellence for Psychotraumatology of the University of Konstanz, the Bodensee-Institut für Psychotherapie, and the NGO “vivo international.” The project aims at integrating refugees between 14 and 22 years in the existing health care system to provide them with mental health services (for more details see Appendix). The project and current study were approved by the Ethics Committee of the University of Konstanz.

The screening took place from March 2020 to January 2022. Government authorities working with refugees in the German state of Baden-Württemberg, mainly near the city of Konstanz, were informed of the upcoming research project. Social workers employed at the refugees’ accommodation informed potential participants between 14–22 years about the project. If refugees agreed to participate, an appointment was scheduled *via* the social worker. Most of the screenings took place in the refugees’ accommodation in a separate office and 8.8% came to the office of the Center of Excellence for Psychotraumatology at the University of Konstanz. The questionnaires were administered in a semi-structured interview by trained psychologists with a PhD or master’s degree working at the Center of Excellence for Psychotraumatology. Trained interpreters were present in 55% of all cases whereas the other interviews took place in English or German. Prior to screening, participants were informed about the study and signed written informed consent. For the *n* = 14 minors a legal guardian gave additional written consent. It was explained that the participation was voluntary, that it would not influence the asylum procedure and that all data was handled confidentially. Moreover, participants were informed that no monetary compensation was offered and that they would not have to pay for a potential and voluntary treatment. When the screening interview, which lasted about 45 min, was completed, the refugees’ symptoms and potential treatment offers were discussed, which included psychotherapy within the project or a transfer to other institutions, for example to refugee psychosocial consultations.

### Measurement instruments and measures

The screening interview included **sociodemographic information** such as age, country of origin, years of education, residence status, health insurance, arrival date in Germany, whether the participants had arrived accompanied by family members and whether they had family living in Germany. Moreover, they were asked if they had been involved in physical fighting since entering the country. In addition to sociodemographic information, **“length of stay”** was calculated according to the arrival date in Germany and **“pandemic months”** was calculated according to the months passed since March 2020.

**Severe physical abuse in childhood** was assessed with the question: “Have you been hit so badly before the age of 15 that you had to go to hospital or needed medical attention?.” Participants responded with a yes/no answer. This question refers to the findings of Webb et al. (2017), who analysed hospital admissions in a national cohort study and showed that having experienced interpersonal violence causing hospitalization prior to the age of 15 years considerably increases the risk of harming one-self and/or being convicted because of a violent crime between the age of 15 to 35 years.

**Emotional distress** was examined using the **Refugee Health Screener** (RHS), a screening tool assessing emotional distress of refugees (Hollifield et al., 2013). The RHS has been applied in various settings with refugees and good reliability and concurrent and predictive validity were found (Hollifield et al., 2013, 2016; Kaltenbach et al., 2017). The RHS-13, which has 13 items, was used in this study (Hollifield et al., 2016). This instrument showed acceptable psychometric properties while being efficient without compromising specificity or sensitivity (Hollifield et al., 2016). The RHS-13 was found to be more economical and valid than the RHS-15 (Borho et al., 2022). Participants rated how much they were bothered by each symptom in the last month, e.g., “feeling down, sad, or blue most of the time.” All items were rated on a 5-point Likert scale ranging from 0 (not at all) to 4 (extremely). The cumulative score ranges between 0–52. Participants (*n* = 5) that did not rate > 10% of the RHS-13 items were excluded from the analyses (Hollifield et al., 2016). If participants’ missing items accounted for ≤10% of the RHS-13 the respective items were set to zero (Kaltenbach et al., 2017). A dichotomous cut-off score was used and psychotherapy was offered to participants with a total score ≥ 11, which indicated high emotional distress (Hollifield et al., 2016). In the present study, Cronbach’s alpha for the instrument resulted in α = 0.91.

The **Integration Index** was developed by the Immigration Policy Lab (IPL) and assesses psychological, economic, political, social, linguistic and navigational dimensions of **integration** (Harder et al., 2018). In this study the IPL-12 scale was used, where each dimension is assessed with two items (Harder et al., 2018). Each of the 12 items can score between 1 and 5 points resulting in a cumulative score between 12 and 60. The cumulative score can be rescaled to range between 0 and 1 for a standardized IPL Integration Index score. Harder et al. (2018) concluded that the questionnaire was appropriate across different countries and different immigrant groups. The Integration Index distinguished among groups of different integration levels and correlated with length of stay and residence status (Harder et al., 2018), showing construct validity. Aligning with the handling of missing items of the RHS-13, participants (*n* = 9) that did not rate > 10% on the IPL-12 were excluded from the analyses and if participants’ missing items accounted for ≤10% of the IPL-12 the respective items were set to one. In this study, the Cronbach’s alpha for the instrument resulted in α = 0.79.

### Data analysis

Version 28 of the Statistical Package for Social Sciences (SPSS; IBM Deutschland GmbH, Ehningen, Germany) was used for all data preparation and statistical analyses. For each analysis the respective level of significance (α ≤ 0.5) is indicated and all correlations were tested two-sided. All sum scores and parameters were generated according to the guidelines of the questionnaires. The requirements for each analysis were examined and if they were not met another suitable analysis was performed.

Kendall’s tau correlations and multiple hierarchical regression analyses served to delineate the contribution of various post-migration stressors and, in particular, the impact of severe physical abuse in childhood on emotional distress and integration. The first multiple hierarchical regression analysis targeted emotional distress and included the variables gender, age, residence status and “pandemic months” as additional variables and severe physical abuse in childhood was entered in the second block. The second multiple hierarchical regression analysis predicted integration with gender, age, length of stay in Germany and emotional distress first entered. Severe physical abuse in childhood was entered in a second block. The model fit of the regressions was examined by assessing in which model the Akaike Information Criterion (AIC) was lowest (Akaike, 1987).

## Results

### Participants’ current living situation

Of the study sample (*N* = 80; mean age 19.7 years; *SD* = 2.2 years), *n* = 14 had entered Germany as accompanied minors and *n* = 24 had entered Germany as an unaccompanied minor. Most participants (60.8% of *n* = 79 participants) did currently not have parents or siblings living in Germany. Most of the participants were from Afghanistan (22.5%), Syria (21.3%), Gambia (16.3%) and Guinea (10%). The years of education ranged from 0 to 15 years and participants had visited on average 7.7 years of education (*SD* = 3.9 years). From all disclosures on residence status (*n* = 78), 24.4% had a residence permit, thus enjoyed a secure residence status, while the asylum process was still in progress for 34.6%, which meant only partly insecurity for these participants. For 41% the residence status was not secure, as their asylum application had been rejected or they were awaiting a result in a follow-up asylum procedure. Regarding their occupation (*n* = 79), 41.8% of participants were engaged in school education or vocational training, 19% in language courses, 29.1% had no occupation, and 10.1% were employed full- or part-time. Sixteen participants reported to have been involved in physical fights since their arrival in Germany.

Experiencing restrictions during the **Covid-19 pandemic** was considered an additional burden. Relative to the length of stay in Germany of an average of 2.5 years (*SD* = 2.0 years; range 2 to 97 months), on average 10.6 months (*SD* = 5.1; range 5 to 23) had passed since the beginning of the Covid-19 pandemic in March of 2020. Of *n* = 51 participants, 34 did not have a health insurance card, hence no direct access to the German health system.

One of the main areas of interest of this study was the mental health status of the present refugee sample, described by their **emotional distress, integration** and having experienced **severe physical abuse in childhood.** As per the RHS-13, emotional distress ranged from 0 to 45, with an average of 16.1 points (*SD* = 12.8). About half of the participants (55.9%) scored above the cut-off of 11. As per the IPL-12, the Integration Index ranged from 14 to 55 with an average score of 30.2 (*SD* = 8.6), the average of the rescaled integration score was 0.38 (*SD* = 0.18). Regarding experiences of severe physical abuse in childhood, *n* = 28 responded that they had experienced severe physical abuse in childhood.

### Contribution of living situation and severe physical abuse in childhood to emotional distress and integration

Table 1 summarizes all correlations of the relevant variables. Emotional distress correlated negatively with integration (*r* = −0.25, *p* = 0.002), positively with severe physical abuse in childhood (*r* = 0.34, *p* < 0.001) and pandemic months (*r* = 0.19, *p* = 0.021). Integration correlated negatively with pandemic months (*r* = −0.23, *p* = 0.005) and positively with length of stay (*r* = 0.48, *p* < 0.001).

**Table 1:** Kendall’s tau correlations.

	Variables	1	2	3	4	5	6	7	8	9
1.	Emotional distress	–								
2.	Integration	−0.247**	-							
3.	Severe physical abuse in childhood	0.342**	0.159	-						
4.	Pandemic months	0.185*	−0.224**	0.155	-					
5.	Length of stay	−0.134	0.479**	0.091	−0.290**	-				
6.	Gender	−0.150	0.010	−0.178	0.233*	−0.130	-			
7.	Age	0.012	0.046	0.045	−0.237**	0.160	−0.304**	-		
8.	Residence status	−0.061	−0.105	−0.176	−0.135	−0.158	−0.079	0.164	-	
		*n* = 78	*n* = 78	*n* = 78	*n* = 78	*n* = 78	*n* = 78	*n* = 78		
9.	Family in Germany	−0.189*	−0.112	−0.379**	0.133	−0.176	0.446**	−0.344**	−0.097	-
		*n* = 79	*n* = 79	*n* = 79	*n* = 79	*n* = 79	*n* = 79	*n* = 79	*n* = 77	

*p < 0.05, **p < 0.01. If values do not depict the full sample (*N* = 80) the deviating number of *n* is indicated.

The first hierarchical regression analysis (see Table 2) showed that **emotional distress** was best predicted by the length of pandemic months (*ß* = 0.46, *p* < 0.001). In the second model, severe physical abuse in childhood added variance (*ß* = 0.31, *p* = 0.005) in addition to the pandemic months (*ß* = 0.38, *p* < 0.001). The latter model (*F* (5, 72) = 5.63 and *p* < 0.001) showed the lowest AIC, explained most variance (adjusted *R*^2^ = 0.23) and the change in *F* (8.564) was significant *p* = 0.005.

**Table 2:** Regression models of emotional distress on gender, age, residence status, pandemic months, and severe physical abuse in childhood.

Model	Predictor	*b*	*SE b*	*β*	*T*	*P*	*R* ^2^	*AIC*
1.	(constant)	−6.100	13.861		−0.440	0.661	0.151	389.015
	Gender	−5.482	4.919	−0.124	−1.114	0.269		
	Age	0.611	0.646	0.103	0.945	0.348		
	Residence status	−0.396	1.713	−0.025	- 0.231	0.818		
	Pandemic months	1.136	0.277	0.456	4.100	<0.001		
2.	(constant)	−6.114	13.195		−0.463	0.664	0.231	382.249
	Gender	−2.698	4.778	−0.061	−0.565	0.574		
	Age	0.461	0.617	0.078	0.747	0.458		
	Residence status	0.453	1.656	0.028	0.247	0.785		
	Pandemic months	0 0.937	0.272	0.376	3.440	<0.001		
	Severe physical abuse in childhood	8.233	2.813	0.312	2.926	0.005		

The determination coefficient *R*^2^ depicts the adjusted *R*^2^ for the respective regression model. AIC = Akaike Information Criterion. These values do not depict the full sample *n* = 78.

**Integration** (see Table 3) was best predicted by length of stay in Germany (*ß* = 0.67, *p* < 0.001) and emotional distress (*ß* = −0.19, *p* = 0.025). Severe physical abuse in childhood (*ß* = 0.21, *p* = 0.019) added variance in the second model in addition to emotional distress (*ß* = −0.28, *p* = 0.002) and length of stay (*ß* = 0.62, *p* < 0.001). Altogether the latter model (*F* (5, 74) = 18.48, *p* < 0.001) showed the lowest AIC, explained 53% of variance and the change in *F* (5.718) was significant *p* = 0.019.

**Table 3:** Regression models of integration on gender, age, length of stay, emotional distress, and severe physical abuse in childhood.

Model	Predictor	*b*	*SE b*	*β*	*t*	*P*	*R* ^2^	*AIC*
1.	(constant)	39.929	6.612		5.585	<0.001	0.495	294.996
	Gender	0.557	2.311	0.021	0.241	0.810		
	Age	−0.598	0.029	−0.153	−1.798	0.076		
	Length of stay	0.236	0.029	0.670	8.125	<0.001		
	Emotional distress	−0.126	0.055	−0.188	−2.290	0.025		
2.	(constant)	36.675	6.414		5.718	<0.001	0.525	291.042
	Gender	1.174	2.257	0.043	0.520	0.604		
	Age	−0.577	0.323	−0.148	−1.789	0.078		
	Length of stay	0.217	0.029	0.617	7.446	<0.001		
	Emotional distress	−0.190	0.060	−0.282	−3.178	0.002		
	Severe physical abuse in childhood	3.838	1.605	0.213	2.391	0.019		

The determination coefficient *R*^2^ depicts the adjusted *R*^2^ for the respective regression model. AIC = Akaike Information Criterion. These values depict the full sample *N* = 80.

## Discussion

The present study addressed the impact of severe physical abuse in childhood on emotional distress and integration of adolescent refugees in Germany during the Covid-19 pandemic. Results confirmed that **severe physical abuse in childhood** explained **emotional distress** to a large extent, even when controlling for gender, age and postmigration factors. This finding is in line with results highlighting the detrimental role of physical abuse in childhood on mental health (Kendall-Tackett, 2002; Margolin and Vickerman, 2011; Lindert et al., 2014; Kalia and Knauft, 2020). Stressful and violent life experiences during the emotional and cognitive development in childhood and adolescence are known to impair brain and endocrine development, and thereby increase vulnerability when subsequent stressors occur (Charmandari et al., 2003; Elbert et al., 2006; Teicher et al., 2016). According to a recent review such adverse childhood experiences might affect emotion regulation and cause cognitive distortions, negative core beliefs and anxiety sensitivity, which in turn increase the risk of trauma-related disorders (Panagou and MacBeth, 2022).

In this research, age was not associated to refugee’s emotional distress. Research (Bean et al., 2007) that found an association between age, traumatic experiences and emotional distress examined a younger sample of unaccompanied refugee minors and their overall traumatic experiences. The negative correlation between emotional distress and having family in Germany supported evidence that being in the host country with family was associated with less emotional distress in the refugees (Laban et al., 2005; Chen et al., 2017; Miller et al., 2018). Two reviews reported a higher prevalence of mental health problems in unaccompanied refugee minors than in those accompanied by family (El Baba and Colucci, 2018; Kien et al., 2019). Family-related emotions and cognitions such as missing the family, loneliness and worries about family in the home country can contribute to the risk for psychopathological developments (Laban et al., 2005). Family was identified as a key domain for interference of post-migration stressors with trauma-related treatment of refugees (Bruhn et al., 2018).

The present study explored the **Covid-19 pandemic** as a further potential stressor which impacts refugee’s mental health and integration. Indeed, results indicated that refugees were more emotionally distressed and less integrated the more “pandemic months” they had experienced since March 2020. “Pandemic months” explained a substantial amount of variance of emotional distress, even after severe physical abuse in childhood was added as a predictor. This emphasizes the Covid-19 pandemic as a stress factor impairing mental health (Alpay et al., 2021; Taquet et al., 2021). When Germany implemented contact restrictions and social distancing in March 2020, helpline contacts increased dramatically, mainly driven by mental health issues such as loneliness, fear and depression (Armbruster and Klotzbücher, 2020). It is possible that the harmful impact of social distancing affected the vulnerable group of refugees even harder, increasing the likelihood that they experienced isolation, loss of control and repeated helplessness (Aragona et al., 2020; Kizilhan and Noll-Hussong, 2020; Mattar and Piwowarczyk, 2020; Rees and Fisher, 2020; Alpay et al., 2021; Gibson et al., 2021). In line with this assumption, more refugees (55.9%) met the cut-off of the RHS-13 for high emotional distress in our research than in prior studies (23–41%, 59, 60). Moreover, Kaltenbach et al. (2017) reported a lower average RHS score in a refugee sample from 2016 (*M* = 11.55, *SD* = 11.92) that lived in Germany for less time (*M* = 6.53, *SD* = 2.99 months) than the present sample. While the higher emotional distress in this sample may be attributable to differing demographic factors, it seems likely that the increased emotional distress could be due to Covid-19 related stressors. This highlights an urgent need to further examine the impact and mechanisms of Covid-19 related stressors.

The present results confirmed that refugees with higher **integration** scores showed less emotional distress – and vice versa (Berry, 2005; Behrens et al., 2015; Han et al., 2016). In a previous study, integration has been shown to increase resilience in unaccompanied refugee minors (Rodriguez and Dobler, 2021). Among factors influencing successful integration, the cultural distance between refugees and the host country has been emphasized. In Norway, less integration and more psychological distress was found in immigrants from non-Western compared to immigrants from Western countries (Dalgard and Thapa, 2007). In the present sample, most of the refugees were from non-Western countries, so that cultural distance might have contributed to distress and poor integration. Harder et al. (Harder et al., 2018) reported average standardized IPL-12 scores of 0.8. in a stratified sample of high-income immigrants, 0.55 in a sample of low-income immigrants, 0.46 in immigrants recently enrolled in English language classes in the United States, and 0.69 in a stratified sample of immigrants in Germany. The average rescaled integration score in our study was lower (0.38), indicating that refugees’ integration levels differ from immigrants’ integration level. Potential differences between refugees and immigrants could be differences in the experience of pre-migration traumatic events, voluntariness of migration and/or abilities to return to their home country (Phillimore, 2011).

A recent review on post-migration stressors and mental health in refugees concluded that length of the asylum procedure -especially a protracted asylum process- was one of the most frequently mentioned post-migration stressors (Gleeson et al., 2020). Yet, **residence status** was neither associated with higher emotional distress (Chen et al., 2017; Gleeson et al., 2020) nor integration (Schick et al., 2016). This suggests that residence status might be associated with other post-migration stressors, such as different living arrangements, different abilities to seek work and a differing access to health care services between asylum seekers and refugees (Toar et al., 2009; Bauhoff and Göpffarth, 2018).

When **severe physical abuse in childhood** was entered into the regression on **integration,** the regression weight of emotional distress increased. In turn, the association between severe physical abuse in childhood and integration became significant, when controlling for emotional distress. It is tempting to assume that severe physical abuse in childhood and emotional distress acted as reciprocal suppressor variables in this multiple linear regression both increasing the other’s regression weight (Conger, 1974). Severe physical abuse in childhood was positively associated with integration. This might indicate that the experience of severe physical abuse in childhood had some adaptive consequences for the refugees. Refugees who have experienced severe physical abuse and other traumatic experiences in their childhood might feel less connected to their home country and may therefore have a stronger motivation to integrate into the host country, than refugees without these experiences. Another explanation has been provided by Haer et al. (2021), who found that social capital increased in communities in the aftermath of experiencing severe violence if the individuals did not develop mental health problems from these experiences. Moreover, research has shown that prenatal stress epigenome-wide interactions with the postnatal environment may enhance resilience in children (Serpeloni et al., 2019). It is possible that the refugees in this research who had experienced severe physical abuse in childhood differed regarding their pre- and postnatal environment, resilience and/or emotion regulation, which in turn resulted in different emotional distress and integration levels.

Mental health of refugees is crucial for their successful integration (Phillimore, 2011; Bakker et al., 2014). Nevertheless, only a small percentage of adolescent refugees with clinically relevant symptoms receive treatment, and in most countries, refugees have only limited access to health care resulting in an unmet need for mental health care (Laban et al., 2007; Silove et al., 2017; Munz and Melcop, 2018; Müller et al., 2019). Munz and Melcop’s (2018) survey on refugees’ healthcare in 14 European countries concluded that refugees often have to wait for long periods before getting treatment and that the care-giving staff, professionals and/or interpreters are often not sufficiently trained. In Germany, language, navigational, cultural as well as structural barriers aggravate the access of refugees to the regular health care system (Adorjan et al., 2017; Schneider et al., 2017). Politics and research should concentrate on understanding and preventing a vicious cycle between poor mental health resulting from pre- and peri-migration traumatic experiences aggravated by post-migration stressors (Walther et al., 2020).

### Limitations

The present sample was culturally diverse with participants originating from 17 different countries. Moreover, the participants faced many different living situations in Germany and showed great heterogeneity regarding their residence status and current activity. Though heterogeneity is a well-known feature of refugee samples (Stenmark et al., 2013; Hecker et al., 2018), diversity may limit general conclusions. In the present study, about half of the interviews needed interpreters; moreover, language- and culture-validated questionnaires were not available for every participant. Nevertheless, the RHS was translated into many different languages while taking into consideration cultural aspects (Hollifield et al., 2016). Furthermore, the interpreters were trained for translating in the mental health context and evidence suggested that the use of professional translators in clinical settings reinforces high-quality psychiatric care (Bauer and Alegría, 2010). Severe physical abuse in childhood was only assessed with one question measuring intensity but not frequency, place, nor perpetrator. Moreover, we did not assess psychological, verbal, or sexual abuse or other traumatic experiences, even though these are also likely to impact refugee’s emotional distress. Hence, the isolated impact of distinct forms of violence during childhood in refugee populations could not be analysed. Future research might further evaluate the impact of severe childhood violence experienced within or outside of the family, and how that might differentiate between accompanied and unaccompanied refugees. The high percentage of male participants in this sample may have limited the generalizability of the results. Hence, in the regression analyses it was controlled for potential gender differences and including gender as a covariate did not influence the associations between emotional distress, integration and the predictor variables. Moreover, German statistics on asylum seekers reported a similar high amount of male refugees in the age groups of 16 to 25 years of 73.1–75.9% (Bundesamt für Migration und Flüchtlinge (2022). General limitations include the retrospective data collection, prone to weakening the reliability because of retrospective memories. All findings depended on self-report questionnaires and the answers were subjective and not verifiable. Furthermore, the study was cross-sectional.

## Conclusion

The impact of the experience of severe physical abuse in childhood on adolescent refugees’ later emotional distress and integration was supported by this study. As expected, experiencing severe physical abuse in childhood led to a higher emotional distress in refugees and higher emotional distress led to a lower integration. These findings highlight the need to take these experiences into consideration during treatment and integration of refugees. Furthermore, this study emphasizes the devastating impact of the Covid-19 pandemic on psychological well-being and successful integration of adolescent refugees in Germany. These results underline the necessity to mitigate the negative psychological consequences of Covid-19 related preventive measures associated with isolation.

## Data availability statement

The raw data supporting the conclusions of this article will be made available by the authors, without undue reservation.

## Ethics statement

The studies involving human participants were reviewed and approved by Ethics Committee of the University of Konstanz. Written informed consent to participate in this study was provided by participants themselves and for minor participants by the participants’ legal guardian/next of kin.

## Author contributions

All authors designed the study. KD, BR, and MS were responsible for acquisition of funding. FP and KD collected the data. FP performed the statistical analyses and drafted the paper under supervision of AC and BR. All authors contributed to the article and approved the submitted version.

## Funding

This research was supported by the Department of Social Affairs Baden-Württemberg (Ministerium für Soziales und Integration Baden-Württemberg), and the costs were covered by the Baden-Württemberg foundation.

## Conflict of interest

The authors declare that the research was conducted in the absence of any commercial or financial relationships that could be construed as a potential conflict of interest.

## Publisher’s note

All claims expressed in this article are solely those of the authors and do not necessarily represent those of their affiliated organizations, or those of the publisher, the editors and the reviewers. Any product that may be evaluated in this article, or claim that may be made by its manufacturer, is not guaranteed or endorsed by the publisher.

## Footnotes

^1^Being aware that refugees and asylum seekers refer to populations with different characteristics, to simplify the terminology we refer to both as refugees.

## Appendix

### Research project

All data was collected in a research cooperation project called “Furchtlos” (fearless) by the Center of Excellence for Psychotraumatology of the University of Konstanz, the Bodensee-Institut für Psychotherapie, and the NGO “vivo international.” “Furchtlos” aims to establish mental health care for refugees between 14 and 22 years of age in the existing health care system. The aim is to improve educational prerequisites as well as social, vocational and societal integration by improving the mental health of young refugees in Baden-Württemberg. Refugees were screened; emotionally distressed refugees were comprehensively informed about their symptoms and they were offered psychotherapy within the project or referred to other institutions. Further, interpreters were trained in translating in the mental health context and served as peer counsellors to support the refugees with therapy, including tasks such as explaining differences of the German health care system, reminding the refugees of therapy appointments or accompanying them to therapy when necessary. Psychologists were trained in Narrative Exposition Therapy (NET) and in Forensic Offender Rehabilitation NET (FORNET), an adaption of NET for perpetrators with a low threshold for aggression (Schauer et al., 2011; Elbert et al., 2012). Furthermore, if needed, the supervisors of the psychologists were trained in NET and FORNET. The treating psychologists decided which treatment was most adequate for their patient, often including NET or FORNET. The overall aim of this proof-of-concept project is the implementation of a proven and established training curriculum for a treatment program specifically tailored to the challenges and needs of young refugees. It is sought to demonstrate a successful model of implementation across the German health care structures. By addressing the prospective therapists, their supervisors, the translators and peer counsellors it is sought to develop structures for stepwise assistance and treatment, including identification, screening, diagnosis, treatment and follow-up.
